# StimuliApp: Psychophysical tests on mobile devices

**DOI:** 10.3758/s13428-020-01491-4

**Published:** 2020-10-09

**Authors:** Rafael Marin-Campos, Josep Dalmau, Albert Compte, Daniel Linares

**Affiliations:** 1 Institut d’Investigacions Biomèdiques August Pi i Sunyer (IDIBAPS), Barcelona, Spain; 2 Centro de Investigación Biomédica en Red de Enfermedades Raras (CIBERER), Barcelona, Spain; 3 Hospital Clinic, University of Barcelona, Barcelona, Spain; 4 Catalan Institution for Research and Advanced Studies (ICREA), Barcelona, Spain; 5 Department of Neurology, University of Pennsylvania, Philadelphia, PA USA

**Keywords:** Open source software, Psychophysics, Stimuli, Mobile, Smartphone, Tablet

## Abstract

Psychophysical tests are commonly carried out using software applications running on desktop or laptop computers, but running the software on mobile handheld devices such as smartphones or tablets could have advantages in some situations. Here, we present StimuliApp, an open-source application in which the user can create psychophysical tests on the iPad and the iPhone by means of a system of menus. A wide number of templates for creating stimuli are available including patches, gradients, gratings, checkerboards, random-dots, texts, tones or auditory noise. Images, videos and audios stored in files could also be presented. The application was developed natively for iPadOS and iOS using the low-level interface Metal for accessing the graphics processing unit, which results in high timing performance.

## Introduction

Psychophysical tests that present stimuli and record responses accurately are essential for studying perception and cognition. They are commonly carried out using software applications running on desktop or laptop computers, but running the software on mobile handheld devices such as smartphones or tablets could be advantageous in some situations. First, their small size and weight could facilitate their use outside the laboratory in places such as clinical environments (Kalia et al., 2014; Bastawrous et al., 2015; McKendrick, Chan, Vingrys, Turpin, & Badcock, 2018; Linares et al., 2020). Second, their touchscreen interface could facilitate their use by people with little experience with more traditional computers. Third, the large number of people owning them, and the ease of installation of applications from common repositories for these devices, could help in the collection of data online. Fourth, they are generally less expensive than desktop or laptop devices.

Psychophysical tests have been carried out on mobile devices with custom applications that implement specific tests such the assessment of visual acuity (Black et al., 2013), contrast sensitivity (Dorr, Lesmes, Lu, & Bex, 2013; Rodríguez-Vallejo, Remón, Monsoriu, & Furlan, 2015; Kollbaum, Jansen, Kollbaum, & Bullimore, 2014), chromatic contrast sensitivity (Bodduluri, Boon, Ryan, & Dain, 2018) or stereoacuity (Rodríguez-Vallejo, Ferrando, Montagud, Monsoriu, & Furlan, 2017), or using an application that allows the sequential presentation of images created offline (Turpin, Lawson, & McKendrick, 2014; McKendrick et al., 2018; Nguyen et al., 2018). To our knowledge, there are no software applications for mobile devices that allow the flexible generation of stimuli to create a wide range of psychophysical tests, and this motivated us to create StimuliApp, an open-source application developed natively for iPadOS and iOS, in which the user can create psychophysical tests by means of a system of menus.

## Development

StimuliApp (www.stimuliapp.com) is a custom application developed natively for iPad and iOS in XCode (version 11.5). XCode is an integrated development environment (IDE) for developing software using the programming language Swift (version 5). Swift is a general-purpose programming language built using the high-performance and open-source LLVM compiler technology, which transforms Swift code into optimized native code.

Visual stimuli with the exception of texts (which are rendered by the Swift library) are rendered using a function written in Metal. Metal is a low-level hardware-accelerated 3D graphic and computer shader application programming interface (API) based on C++14. Similar to OpenGL, it uses the graphics processing unit (GPU) of the device to perform the calculations in parallel.

Auditory stimuli, taking advantage of the versatility of Swift to implement C code, are coded directly in C for better performance. They are generated at a high audio rate of 44.1 kHz.

Touch information is sampled at 120 Hz in all devices except for the iPad Pro 11-inch first generation (and later) and the iPad Pro 12.9-inch third generation (and later), in which the sampling rate is 240 Hz.

## Graphical user interface (GUI)

**Fig. 1:**
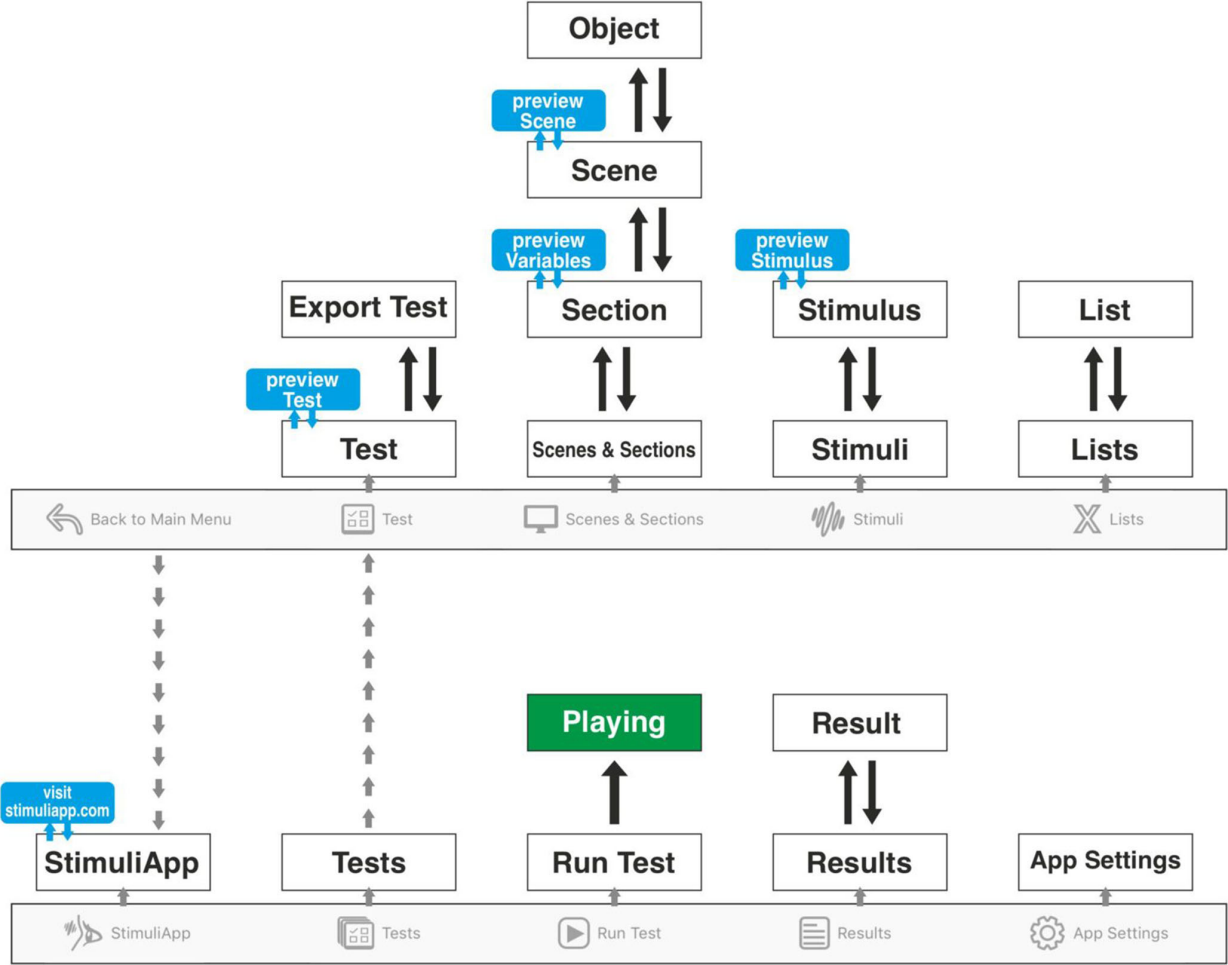
Hierarchy of menus in StimuliApp

To generate a psychophysical test, StimuliApp uses a graphical user interface (GUI) consisting of a system of menus (Fig. 1). Each test is a collection of sections. A section, for example, could be the instructions of the test or each of the trials to be presented. Each section will be a sequence of scenes. For example, the *First trial* section could have the scenes Fixation, Target and Feedback. Each scene could include several stimuli. For example, the *Target* scene might consist of the simultaneous presentation of two gratings and one sound.

StimuliApp incorporates several demonstration tests. Tutorials of how these tests were built can be found at www.stimuliapp.com. To make a copy and modify a demonstration test (or any test created by the user), it is necessary to perform a long tap on the name of the test in the *Test* menu.

To facilitate the interactive creation of a test, each stimulus, scene or section can be independently previewed. When previewing a scene, it is possible to advance it frame by frame, which could be particularly useful for monitoring rapidly changing stimuli.

Once a test is created, it can be run in the same device by going to the *Run test* menu. It can also be exported to any other device with StimuliApp installed (for example, a test created on an iPad could be run on an iPhone). To export a test, *Export test* should be pressed in the *Test* menu. The user will be able to email a *.stimulitest* file containing a *.json* description of the parameters of the test to any device. To import the test in the receiver device, the *.stimulitest* file should be opened with StimuliApp (if StimuliApp does not appear in the list of applications, the user should click the *More* option).

The *.stimulitest* files, as they contain a .*json* description, can be edited with any text editor. By default, the files are generated in a single line, but some editors (e.g. atom) have an automatic option to change it to multiple lines. There are also online tools (e.g. JSON Formatter) to change a single line to multiple lines. We think, however, that it is easy to get lost in the structure of a .*json* file and recommend the modification of tests within StimuliApp.

Once a test is run, the results can be accessed in the *Results* menu. There, the user will find two text files that can be sent by email. One file—with *txt* extension—includes information about the settings such as audio rate, screen resolution or frame rate. The other file—with *csv* extension—consists of a table in which each column is a variable of the test and each row is a section (trial). As this is a typical structure for data analysis, the *csv* file can be read by standard software to that end such as R, Python, Excel or SPSS.

## Stimuli

**Fig. 2:**
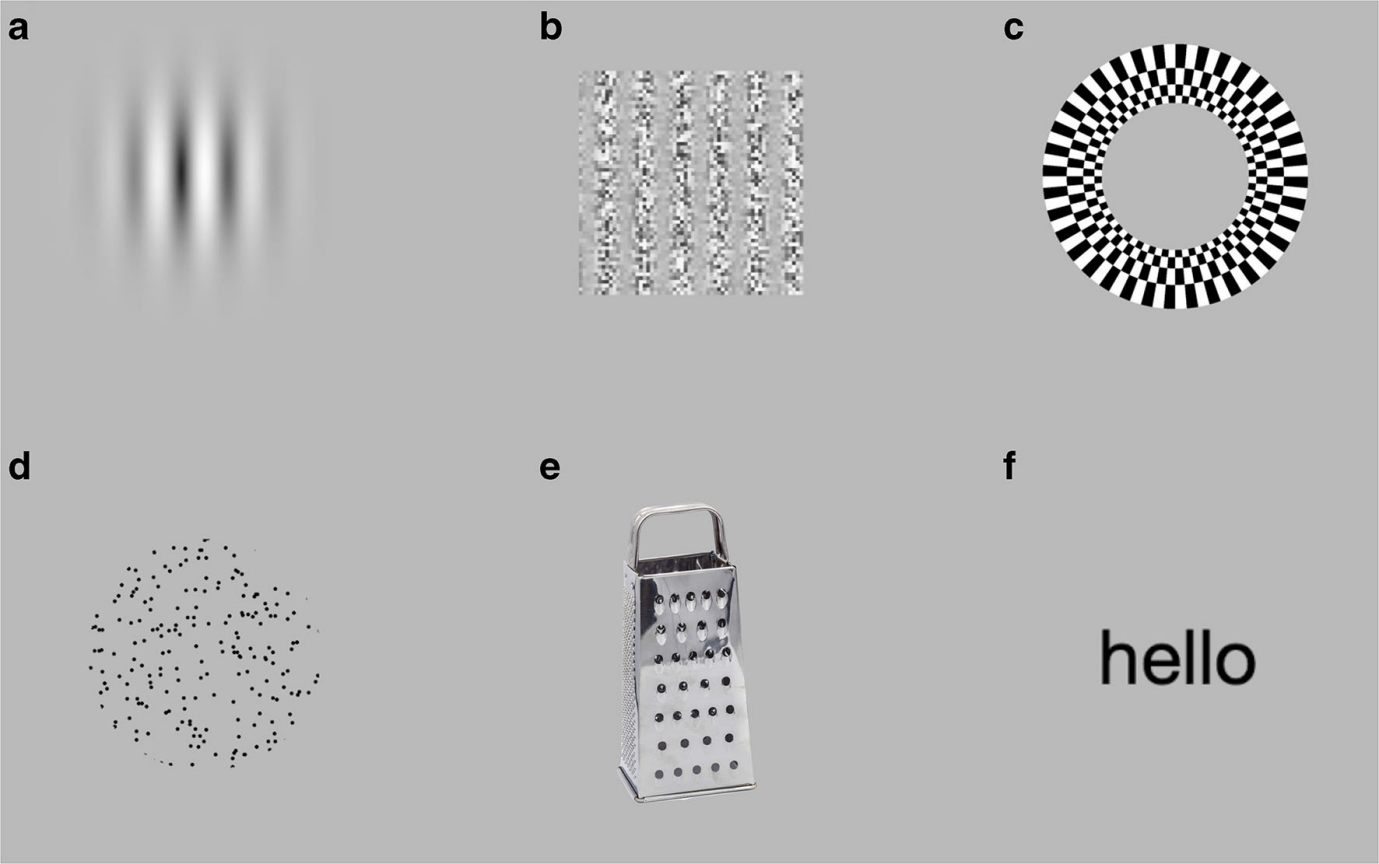
Examples of stimuli that can be presented using StimuliApp. (A) Gabor. (B) Grating with modulated carrier contrast. (C) Radial checkerboard. (D) Random-dots. (E) Image from a file. (F) Text

StimuliApp offers a large number of templates for creating stimuli commonly used in the study of perception and cognition (Fig. 2; Lu & Dosher, 2013) including patches, gradients, gratings (Gabors), checkerboards (rectangular or radial), random-dots (linear, radial, expansive), texts, tones or auditory noise. Stimuli can also consist of images, videos and audios stored in files.

Each stimulus has specific properties that define the type of stimulus and general properties such as duration, position, orientation, size, shape, noise filter, color or contrast, which are common. An interesting feature of StimuliApp is that contrast can be manipulated quasi-continuously as the noise-bit method is implemented (Allard & Faubert, 2008). All properties can be modified dynamically in the course of the test.

The values of the properties can be specified independently for each stimulus in different units. Sizes and distances, for example, can be specified in pixels, centimeters, inches, or degrees of visual angle, which are calculated taking into account the viewing distance specified by the user and the pixel resolution of the screen of the device, which is directly detected by StimuliApp. Time can be specified in frames or seconds. Luminance is specified in fractions of the maximum brightness of the device. Interestingly, the value of the luminance in cd/m^2^ is automatically displayed, since StimuliApp recognizes the model device and incorporates a table with the maximum luminance of each device. The nominal maximum luminance values were retrieved from apple.com and might slightly differ from the displayed values due to variations across series or the time in use of the displays (Caffery, Manthey, & Sim, 2016). Importantly, for luminance—and also color—to be displayed consistently, the technologies Auto-Brightness, True Tone and Night Shift of the device should be disabled in System Preferences.

To facilitate the selection of the values across sections (for example, trials) the method of constant stimuli and several adaptive methods are implemented (Kingdom & Prins, 2016).

## Responses

Responses can be taps, the movement of a finger or the movement of an electronic pencil on the screen. A virtual keyboard can also be displayed or an external keyboard used.

## Timing

To compute the RGB values of the stimuli in real-time, the computation should last less than the duration of a frame (16.67 ms for a refresh rate of 60 Hz and 8.33 ms for 120 Hz, for example). If the computation lasts longer than a frame, the previous image will be on the screen until the computation is finished (dropped frame).

**Fig. 3:**
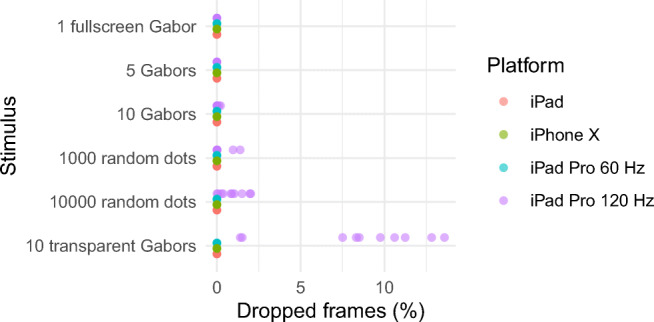
Percentage of dropped frames for different stimuli in different platforms. Each dot indicates the percentage of dropped frames when we displayed the stimulus for 120 s. We presented each stimulus 10 times. The Gabors were 300 × 300 pixels (excepting the fullscreen Gabor). The transparent Gabors had quasi-continuous contrast using the noise-bit method (Allard & Faubert, 2008)

Using the GPU Frame Capture tool and the Metal API validation tool, we calculated the number of dropped frames for several stimulus tests, which we presented 10 times during 120 s on several platforms (iPad 6th generation 2018, iPhone X 2017, iPad Pro 1st generation 10.5 inches 2017 at 60 and 120 Hz). We tried many simple stimuli typically used in visual psychophysics, and all resulted in zero dropped frames in all platforms. We were able to get dropped frames only when we displayed complex stimuli on the iPad Pro at 120 Hz (Fig. 3). All of these stimuli resulted in zero dropped frames in the other platforms or when the iPad Pro was run at 60 Hz (Fig. 3).

When a test is previewed or run, and one or more frames are dropped, a report is generated indicating the duration of the dropped frames. This information could be used to reduce the computational costs of the test.

To assess the accuracy and precision reproducing sounds, we presented 1000 sounds in StimuliApp specifying a duration of 100 ms each. Connecting an output jack to an oscilloscope (Tektronix TDS 1012), we measured the generated durations and found a mean duration of 100 ms and a standard deviation of less than 1 ms.

To assess audiovisual synchrony, we presented 1000 times a visual and an auditory signal of 100 ms of duration specifying the same time onset in StimuliApp. Connecting an output jack and a photodiode to the oscilloscope, we measured the generated onset asynchronies and found that the mean asynchrony depended on the device and was between −10 ms and 10 ms (this value could be specified in StimuliApp to correct it). The standard deviation was less than 1 ms.

## Extending StimuliApp

Developers can add stimuli to StimuliApp by downloading its open source code and compiling it including a new independent Metal function for each new stimulus, and one descriptive class in Swift with the description of its new parameters. These new parameters will be added to the general parameters common to all stimuli such as size, contrast, angle, noise and temporal changes. For more information visit https://github.com/marinraf/StimuliApp.

## Workflow for collecting data using StimuliApp: Examples

The first example describes a situation where the test is created and run on the same device. This possibility could be useful to administer tests in person. An experimenter installs StimuliApp on an iPad and uses the application to create a new test. Once the test is created, the experimenter goes to the *Run* section of the application and hands the iPad to a participant. After the participant finishes the test, the experimenter goes to the *Results* section and sends the two output data files with the results to the email of the experimenter.

The second example describes a situation where the test is created on one device and run on another device. This possibility could be useful to administer tests remotely. An experimenter installs StimuliApp on an iPhone and uses the application to create a new test. Once the test is created, the experimenter exports the test (see GUI section) and sends it by email to the participant. The participant installs StimuliApp on the iPad and then opens the file sent by the experimenter with StimuliApp. Then, the participant goes to the *Run* section and runs the test. After finishing, the participant goes to the *Results* section and sends the two files with the results to the experimenter.

## Tutorial

In this section, we describe how to build a simple test to measure orientation discrimination using the method of constant stimuli (Kingdom & Prins, 2016). Each trial will consist of a fixation cross followed by a grating with a small clockwise or counterclockwise rotation relative to the vertical orientation. The participant will need to tap the right or left side of the screen to indicate clockwise or counterclockwise rotation, respectively. Further tests with more detailed information including screen captures can be found at www.stimuliapp.com.

First, we create a fixation cross:

- Go to the *Test* menu (Fig. 1) and press *new test*.
- Go to the *Stimuli* menu (Fig. 1) and press *new stimulus*.
- Change the stimulus *shape* to *cross*.

Then, we include the fixation as the first scene of a section:

- Go to the *Scenes & Sections* menu (Fig. 1) and press *new section*.
- Within that section, press *new scene*.
- Within the scene, press *new object* and select *stimulus1*.
- Press *durationValue* and change the scene duration to 0.5 seconds.
- Go to the *Test* menu, press *firstSection* and select *section1*.

Then, we create the grating:

- Go to the *Stimuli* menu and press *Stimuli* in the top-left corner to go to the home screen of the *Stimuli* menu. Press *new stimulus*.
- Press *type* and change the stimulus type to *grating*.
- Change the stimulus *shape* to *ellipse*.

Then, we include the grating as the second scene within the section:

- Go to the *Scenes & Sections* menu and press *section1* in the top-left corner to go to the *Sections* menu. Press *new scene*.
- Within the scene, press *new object* and select *stimulus2*.

Then, we tell StimuliApp to present the stimulus multiple times with a different orientation each time:

- Go to the *Stimuli* menu, press the property *gratingRotation* and change it to *variable*.
- Go to the *Lists* menu, press *new list* and select *new list of numeric values.*
- Press *add linear sequence*. For the *First value*, *Last value* and *Number of values* assign the numbers -0.03, 0.03 and 7, respectively (the first two are angles in radians).
- Go to the *Scenes & Sections* menu and press *section1* (in the top-left corner of the screen).
- Press the newly created property *scene2_object1_gratingRotation* and assign the list created above.
- From the *selectionMethod* select *all values in random order*.
- Press *section1* and change the number of repetitions to 20.

Then, we allow the user to make responses:

- Go to *scene2*, press *response* and add a *left or right* response.

Finally, we run the test:

- Go to the *Main menu* and then to the *Run test* menu. Press *Run: test1.*

The text file with the results can be found in the *Results* menu.

## Discussion

StimuliApp is an open-source application that enables the generation of a wide range of psychophysical tests on mobile devices. As it was developed natively for iPadOS and iOS, the supported mobile devices are the iPad and the iPhone. We decided to program the application natively to try to achieve high timing performance, and our measurements suggest that this was achieved for stimulus presentation, as StimuliApp, for most situations, results in zero dropped frames. Future research should also test the temporal precision of touch responses. We chose the Apple ecosystem because it has a relatively small number of available models, which facilitates testing, and because the application, by recognizing the model, can present about the same stimuli independently of the device. The value of the luminance, for example, is directly displayed in cd/m^2^ without the need for a photometer.

For desktop and laptop computers, the flexible generation of psychophysical tests could be performed among others using the platform-independent packages Psychtoolbox (Kleiner, Brainard, & Pelli, 2007) and PsychoPy (Peirce, 2007), both resulting in high timing performance (Bridges, Pitiot, MacAskill, & Peirce, 2020). PsychoPy offers the possibility of generating psychophysical tests using a GUI without the need for coding, similar to StimuliApp. It also allows the execution of psychophysical tests on the web browser, although a decrease in timing performance has been reported (Bridges et al., 2020; Anwyl-Irvine, Dalmaijer, Hodges, & Evershed, 2020). Running PsychoPy on the browser of a mobile device could be an alternative to creating psychophysical tests on mobile devices.

In the Introduction section we described a number of advantages of using mobile devices to run psychophysical tests, but there are also limitations. First, psychophysical tests have traditionally been conducted using CRT monitors, which have better spatiotemporal properties than the LCD screens incorporated in mobile devices (Ghodrati, Morris, & Price, 2015; Elze & Tanner, 2012; Packer et al., 2001). In a previous study, however, we compared motion sensitivity for very brief stimuli presented on an iPad (using an earlier version of StimuliApp) or a CRT monitor (using PsychoPy) and found comparable values (Linares, Marin-Campos, Dalmau, & Compte, 2018). Second, chin rests—which are often used when the experimental set-up consists of an external monitor connected to a desktop computer—could hardly be combined with mobile devices, hindering the maintenance of a constant viewing distance. To ameliorate this issue, in our previous studies (Linares et al., 2018, 2020) the experimenter monitored the participants to ensure that they held the same approximate position during the test. A future solution to this issue could be the use of a facial recognition system like Face ID to measure the viewing distance and alert the participant if it changes during the test. This technology might be also used to track eye movements. Third, many mobile devices incorporate glossy screens, whose glare might be superimposed on the visual stimuli. To minimize this problem, the participant should run the test in a location where reflections are minimized.

Tablets and smartphones are increasingly used to acquire data in behavioural sciences (Miller, 2012; Woods, Velasco, Levitan, Wan, & Spence, 2015). Here, we present an application for iPadOS and iOS devices to create psychophysical tests with high temporal precision.
